# Curcumin and capsaicin regulate apoptosis and alleviate intestinal inflammation induced by *Clostridioides difficile* in vitro

**DOI:** 10.1186/s12941-022-00533-3

**Published:** 2022-09-26

**Authors:** Masoumeh Azimirad, Maryam Noori, Fahimeh Azimirad, Fatemeh Gholami, Kaveh Naseri, Abbas Yadegar, Hamid Asadzadeh Aghdaei, Mohammad Reza Zali

**Affiliations:** 1 Foodborne and Waterborne Diseases Research Center, Research Institute for Gastroenterology and Liver Diseases, Shahid Beheshti University of Medical Sciences, Tehran, Iran; 2 Basic and Molecular Epidemiology of Gastrointestinal Disorders Research Center, Research Institute for Gastroenterology and Liver Diseases, Shahid Beheshti University of Medical Sciences, Tehran, Iran; 3 Gastroenterology and Liver Diseases Research Center, Research Institute for Gastroenterology and Liver Diseases, Shahid Beheshti University of Medical Sciences, Tehran, Iran

**Keywords:** *Clostridioides difficile* infection, Curcumin, Capsaicin, Inflammation, Apoptosis, Tox-S

## Abstract

**Background:**

The dramatic upsurge of *Clostridioides difficile* infection (CDI) by hypervirulent isolates along with the paucity of effective conventional treatment call for the development of new alternative medicines against CDI. The inhibitory effects of curcumin (CCM) and capsaicin (CAP) were investigated on the activity of toxigenic cell-free supernatants (Tox-S) of *C. difficile* RT 001, RT 126 and RT 084, and culture-filtrate of *C. difficile* ATCC 700057.

**Methods:**

Cell viability of HT-29 cells exposed to varying concentrations of CCM, CAP, *C. difficile* Tox-S and culture-filtrate was assessed by MTT assay. Anti-inflammatory and anti-apoptotic effects of CCM and CAP were examined by treatment of HT-29 cells with *C. difficile* Tox-S and culture-filtrate. Expression of BCL-2, SMAD3, NF-κB, TGF-β and TNF-α genes in stimulated HT-29 cells was measured using RT-qPCR.

**Results:**

*C. difficile* Tox-S significantly (*P* < 0.05) reduced the cell viability of HT-29 cells in comparison with untreated cells. Both CAP and CCM significantly (*P* < 0.05) downregulated the gene expression level of BCL-2, SMAD3, NF-κB and TNF-α in Tox-S treated HT-29 cells. Moreover, the gene expression of TGF-β decreased in Tox-S stimulated HT-29 cells by both CAP and CCM, although these reductions were not significantly different (*P* > 0.05).

**Conclusion:**

The results of the present study highlighted that CCM and CAP can modulate the inflammatory response and apoptotic effects induced by Tox-S from different clinical *C. difficile* strains in vitro. Further studies are required to accurately explore the anti-toxin activity of natural components, and their probable adverse risks in clinical practice.

## Background

*Clostridioides* (*Clostridium*) *difficile* is considered to be the leading cause of nosocomial diarrhea with high morbidity and mortality worldwide [1]. *C. difficile* infection (CDI) caused by toxigenic strains leads to complications of the disease ranging from asymptomatic colonization to potentially life-threatening pseudomembranous colitis and toxic megacolon [2–4]. Typically, predisposition to CDI occurs following the disruption of normal gut microbiota during or after broad-spectrum antimicrobials treatment, germination of *C. difficile* spores into vegetative cells and finally toxin production in the intestine [5].

Toxigenic strains of *C. difficile* cause intestinal inflammation with the secretion of two major enterotoxins with cytotoxic properties, toxin A (TcdA) and toxin B (TcdB), that belong to the large clostridial glycosylating toxin (LCGT) family [6, 7]. Both toxins, particularly TcdA, can glucosylate the Rho family GTPases, and thereby result in rearrangement of the actin cytoskeleton leading to cell rounding and apoptosis in intestinal epithelial cells (IECs) [8, 9]. Furthermore, secretion of these toxins into the gastrointestinal tract provokes a complex cascade of cellular events, which results in induction of inflammatory response and loss of epithelial integrity [10–13].

Currently, vancomycin, metronidazole and fidaxomicin are the most recommended anti-CDI antibiotics globally [14, 15]. Despite notable efficacy of vancomycin and metronidazole against *C. difficile,* which are regarded as the first-line therapies especially in developing countries, they are widely associated with high recurrence rate (20–40%) of CDI (rCDI) [16, 17]. In recent years, a new bactericidal narrow-spectrum antibiotic, fidaxomicin, has been introduced as a highly effective option to prevail over the rCDI cases [18, 19]. However, its markedly higher cost compared to conventional anti-CDI antibiotics has limited the widespread use of this antimicrobial agent [20]. Moreover, rapid emergence of hypervirulent *C. difficile* strains and their rising antimicrobial resistance have led to suboptimal clinical outcomes and even resulted in CDI treatment failures globally [21]. Thereby, a plethora of efforts are devoted to explore new promising therapies and more effective preventive interventions to overcome CDI [14]. A growing number of recent studies have focused on pharmacological actions of plant-derived compounds, particularly those included in human diet [22–26]. Moreover, herbal-derived compounds are regarded as safe and less toxic therapeutic candidates, which can target cellular viability of microorganisms and are capable to modulate toxin production in pathogenic microbes [27, 28]. Two well-known plant-derived compounds, curcumin (CCM), and capsaicin (CAP) have frequently been studied for their pharmacological actions [29, 30]. CCM, a natural phytoconstituent extracted from the rhizomes of turmeric (*Curcuma longa*), is found to possess a wide array of biological features including anti-oxidant, anti-tumor and also anti-inflammatory properties [31–33]. Furthermore, CCM and its derivatives, bisdemethoxycurcumin and demethoxycurcumin have been shown to exert antimicrobial activities against different bacterial species particularly multidrug resistant (MDR) strains [34–36]. CAP, an active component obtained from the plants of genus capsicum, is the most abundant pungent molecule produced by chili pepper [30]. Meanwhile, CAP is an agonist of transient receptor potential vanilloid subfamily member 1 (TRPV1), which is a sensor protein that responds to a variety of stimuli [37]. CAP and other capsaicinoids, dihydrocapsaicin and nordihydrocapsaicin have been shown to exert various pharmacological functions, such as anti-oxidant, anti-tumor and also anti-obesity properties similar to CCM [30, 38]. Moreover, CAP has attracted much interest due to its potential antimicrobial properties against various bacterial pathogens [39]. In view of the imperative requirement of new anti-clostridial treatments, we examined the antimicrobial activities of CCM and CAP on three toxigenic *C. difficile* clinical strains (RT 001, RT 126, RT 084) and *C. difficile* ATCC 700057 (RT 038) as non-toxigenic control. Furthermore, we assessed the inhibitory effects of these natural products on toxin-mediated cytotoxicity and apoptosis in Vero and HT-29 cells treated with culture-filtrate and cell-free supernatant (Tox-S) of aforementioned strains.

## Material and methods

### Bacterial strains and growth conditions

The four *C. difficile* strains that were used in this study included non-toxigenic *C. difficile* ATCC 700057 (A^−^B^−^ CDT^−^) and toxigenic clinical isolates of *C. difficile* RT 001 (A^+^B^+^ CDT^+^), RT 126 (A^+^B^+^ CDT^+^), RT 084 (A^+^B^−^ CDT^−^). *C. difficile* strains were obtained from Department of Anaerobic Bacteriology in Research Institute for Gastroenterology and Liver Diseases in Tehran, Iran. All samples were cultured on cycloserine-cefoxitin-fructose agar (CCFA, Mast) supplemented with 7% horse blood under anaerobic conditions of 85% N_2_, 10% CO_2_ and 5% H_2_ (Anoxomat® Gas Exchange System, Mart Microbiology BV) at 37 °C for 48–72 h after an alcohol shock treatment [40].

### Antibacterial agents

Two natural products, CAP and CCM were dominated for investigation based on previously reported evidence in literature, their current popularity, and feasibility. CAP and CCM powders were purchased from Sigma Chemical Co. (USA), and stock solutions of products were prepared in 20% dimethyl sulfoxide (DMSO) as previously described [41]. Prior to use, different concentrations of CAP (10, 20, 50, 75, 128 and 256 μM) and CCM (5, 10, 20, 30, 60, 128 and 256 μM) were freshly prepared in sterile distilled water (SDW). Vancomycin (VAN), metronidazole (MET) and fidaxomicin (FDX) were used as antimicrobial controls in susceptibility testing.

### Preparation of Tox-S and culture-filtrate

The culture-filtrate and Tox-S of *C. difficile* strains were obtained as described previously with slight modification [42]. *C. difficile* strains were cultured on CCFA medium for 48 h, from which suspensions equivalent in turbidity to a 2 McFarland standard were prepared in 0.85% sterile saline. A volume of 100 µl of each suspension was inoculated into a 10 ml pre-reduced brain heart infusion (BHI) broth, and incubated for 5 h under anaerobic conditions. Then, the broth cultures were aseptically sealed and incubated for a further 5 days at 37 °C in a shaking incubator (Labtech, Korea) with shaking at 120 revolutions per minute (rpm). After incubation, cultures were centrifuged at 4000 × g for 5 min and the supernatants were passed through a 0.22 µm-pore size Millipore filter to remove cells and debris. The presence of *C. difficile* toxins A and B in the supernatant of toxigenic strains was evaluated by enzyme-linked immunosorbent assay (ELISA, Generic Assays, Germany) according to the manufacturers’ instructions.

### Determination of minimum inhibitory concentration (MIC)

The MIC for natural products and comparator antimicrobial agents were measured using the broth microdilution method and agar dilution method, respectively as previously described [41, 43]. Briefly, two-fold serial dilutions (0.5 to 256 μM) of each product were prepared across a 96-well plate in pre-reduced Brucella broth supplemented with yeast extract, l-cysteine, 5 µg/ml hemin, 1 µg/ml vitamin K1 and 5% (v/v) lacked sheep blood in triplicate. A bacterial suspension of approximately 10^5^ cfu/mL from each strain was prepared and seeded into the 96-well plates. Plates were then incubated for 48 h at 37 °C under anaerobic conditions. MICs were determined as the lowest concentration of a given drug that suppressed the visual growth of bacteria. The MICs of VAN, MET and FDX as antibiotic controls were determined by agar dilution method according to CLSI criteria (document M100-S28) [44]. The clinical breakpoints for antimicrobial agents used in this study are those previously provided by Peng et al. [45]. A series of two-fold dilutions of each antibiotic with final concentrations ranging from 0.5 to 256 μg/ml for VAN and MET, and 0.5–16 μg/ml for FDX was made in pre-reduced supplemented Brucella agar. For inoculum preparation, test bacterial strains were cultured anaerobically on CCFA plates at 37 °C for 48 h. Then, pure bacterial cultures were suspended in pre-reduced 0.85% saline solution to yield McFarland 0.5 equivalent to a final concentration of approximately 1 × 10^6^ CFU ml^−1^. MICs were determined as the lowest concentration of a given drug at which no visual growth of bacteria was observed after incubation for 48 h at 37 °C in an anaerobic atmosphere. Growth controls were performed by addition of bacterial inoculum into the antibiotic-free medium.

### Cell culture and growth conditions

The Vero (African green monkey kidney) and HT-29 (human colorectal adenocarcinoma) cell lines were obtained from Iranian Biological Resource Center (IBRC). The cells were maintained in high-glucose Dulbecco’s modified Eagle’s medium (H-DMEM, Gibco, USA) supplemented with 10% heat-inactivated fetal bovine serum (FBS), 100 U/ml of penicillin, and 100 μg/ml of streptomycin in a 5% CO_2_ humidified incubator at 37 °C. To prepare confluent monolayers (approximately 80%), Vero and HT-29 cells were dispensed into 96-well trays at 6 × 10^4^ cells/well and 5 × 10^5^ cells/well, respectively. The numbers of viable and dead cells in each treatment were assessed by trypan blue (Sigma, USA) exclusion assay. Briefly, the trypsinized cell suspension was diluted in 1:10 ratio using a 0.025% (w/v) trypan blue solution. Cell counting was performed using a Neubauer Improved Hemocytometer (PerciColor HBG, Germany).

### Cell cytotoxicity assay

Cytopathic effect (CPE) of the toxins was determined by cell rounding assay using Vero cells as described previously with some modifications [46]. Two concentrations (100 and 500 μg/ml) of *C. difficile* culture-filtrate and Tox-S in H-DMEM were prepared with a total volume of 100 µl per well and were added to the Vero cell monolayers, and incubated them for 4 h at 37 °C in 5% CO_2_. The CPE indicated by 90% cell rounding was then determined visually using an inverted microscope (Olympus Corporation, Tokyo, Japan) at × 400 magnification. The experiments were performed in triplicate.

Different concentrations of CAP (10, 20, 50 and 75 μM) and CCM (5, 10, 20 and 30) were freshly prepared in sterile distilled water and were added to the Vero cell monolayers, and incubated for 1, 4, 24 h at 37 °C in 5% CO_2._ Different concentrations of CCM and CAP was not revealed the CPE on Vero cells after 24 h treatment. The experiments were performed in triplicate.

### Cell viability assay

The cell viability of HT-29 cells exposed to different concentrations of natural products, *C. difficile* culture-filtrate, and Tox-S was measured using an MTT assay. Briefly, 5 × 10^5^ cells/well were seeded in 96-well plates and allowed to adhere overnight. Cells were treated with different concentrations of CAP (10, 20, 50 and 75 µM) and CCM (5, 10, 20, 30 and 60 µM) for 24 h. Culture-filtrate and Tox-S (100 and 500 μg/ml) treated cells were incubated for 4 h as described previously [47]. Following incubation, 10 μl of 3-(4,5 dimethylthiazol-2-yl)-2,5-diphenyl tetrazolium bromide (MTT) was added to each well, and the cells were incubated for 4 h at 37 °C in 5% CO_2_. The reaction was then stopped by lysing the cells with 200 μl of dimethyl sulfoxide (DMSO) for 15 min. Monolayers in the growth media were used as negative controls and wells without cells served as blanks. The plates were then incubated at 37 °C for 10 min and the optical density (OD) of each well was measured at 560 nm using a microplate reader (BioTek, USA). The cell viability was calculated using the equation Cell viability (%) = (X × 100%)/Y, where “X” is the absorbance of treated cells and “Y” the absorbance of untreated cells.

### Treatment of HT-29 cells with culture-filtrate, Tox-S, CAP and CCM

The highest concentrations of culture-filtrate (100 μg/ml) and Tox-S (100 μg/ml), and different concentrations of CAP (10, 20, 50 μM) and CCM (10, 20, 30 µM) with no visible effect on cell monolayers were used in this assay. Briefly, HT-29 cells were pre-treated with 100 μg/ml of *C. difficile* culture-filtrate and Tox-S, and incubated for 4 h at 37 °C in 5% CO_2_. After incubation, cells were treated with various over-mentioned concentrations of CAP and CCM for 24 h at 37 °C in 5% CO_2_ incubator. All treatments were run in triplicate. Untreated cells, culture-filtrate from non-toxigenic *C. difficile* ATCC 700057 and Tox-S from three other toxigenic strains without the addition of natural products were used as controls. After treatment, the cells were lysed for RNA extraction and gene expression analysis.

### RNA preparation and cDNA synthesis

Total RNA from treated HT-29 cells was extracted using RNeasy Mini Kit (Qiagen, Germany) following the manufacturer’s protocol. RNA concentration and quality was assessed spectrophotometrically by using NanoDrop spectrophotometer (ND-1000, Thermo Scientific, USA). The RNA samples were frozen at −80 °C until used for cDNA synthesis. The RNA was reverse-transcribed to cDNA using the PrimeScript™ RT Reagent Kit (Takara, Japan) according to the manufacturer’s instructions. All cDNA preparations were frozen at −20 °C until further use.

### Quantitative real-time PCR (RT-qPCR)

The RT-qPCR analysis was performed with the Rotor-Gene® Q (Qiagen, Germany) real-time PCR system using BioFACT™ 2X Real-Time PCR Master Mix (BIOFACT, South Korea). The oligonucleotide primers and amplification conditions used for gene expression analysis of BCL-2, SMAD3, TGF-β, TNF-α, and NF-κB are listed in Table 1. The β-actin housekeeping gene served as the reference gene. To confirm amplification specificity, a melting analysis and subsequent agarose gel electrophoresis were performed after each run. All reactions were run in triplicate. Relative gene expression was calculated by the 2^−ΔΔCt^ method, and the expression levels were given as the fold change relative to the control samples.

**Table 1:** Oligonucleotide sequences used in this study

Target gene	Primer designation	Oligonucleotide sequence (5′-3′)	PCR conditions	Reference
BCL-2	BCL-2	F: GAGCTGGTGGTTGACTTTCTC	95 °C 10 min, 40 cycles (95 °C 10 s; 56 °C 35 s; 72 °C 20 s), 72 °C 5 min	[48]
	BCL-2	R: TCCATCTCCGATTCAGTCCCT		
TNF-α	TNF-α	F: AGCCCATGTTGTAGCAAACC		[49]
	TNF-α	R: TGAGGTACAGGCCCTCTGAT		
TGF-β	TGF-B	F: CAATTCCTGGCGATACCTCAG		[50]
	TGF-B	R: GCACAACTCCGGTGACATCAA		
NF-kB	NF-kB	F: CCAGACCAACAACAACCCCT		[51]
	NF-kB	R: TCACTCGGCAGATCTTGAGC		
SMAD3	SMAD3	F: GCCTGTGCTGGAACATCATC		[52]
	SMAD3	R: TTGCCCTCATGTGTGCTCTT		
β-actin	ACTBF	F: ATGTGGCCGAGGACTTTGATT		[53]
	ACTBF	R: AGTGGGGTGGCTTTTAGGATG		

### Statistical analysis

Statistical analysis was carried out with GraphPad Prism software version 5.04 (Inc., CA, USA). Unpaired student’s t test and one-way analysis of variance (ANOVA) were used to determine the statistical significance between groups. The data were presented as the averages of at least three independent experiments; error bars represent the standard deviations (SD). Differences were considered statistically significant when *P* < 0.05; *P < 0.05, **P < 0.01, ***P < 0.001 and ****P < 0.0001.

## Results

### Antimicrobial susceptibility testing

The MIC of natural products and antibiotic controls examined against the four *C. difficile* strains were presented in Table 2. Both CAP and CCM showed an MIC of 256 μM for *C. difficile* RT 001 and RT 126, while a two-fold lower MIC (128 μM) was detected for *C. difficile* RT 084 and *C. difficile* strain ATCC 700057. In addition, all the four *C. difficile* strains were interpreted to be susceptible against antimicrobial controls tested in this work.

**Table 2:** Interpretive criteria and MIC values of CCM, CAP and antimicrobial agents tested against *C. difficile* strains

Agents	Interpretation			MIC			
	S	I	R	RT 001	RT 126	RT 084	700057
CCM (μM)	–	–	–	256	256	128	128
CAP (μM)	–	–	–	256	256	128	128
MET (μg/ml)	≤ 8	16	≥ 32	1	0.5	0.5	0.5
VAN (μg/ml)	≤ 2	NA	≥ 8	0.5	0.5	1	0.5
FDX (μg/ml)	≤ 1	> 1	NA	0.5	0.5	0.5	0.5

Breakpoints were defined as susceptible (S), intermediately resistant (I), or resistant (R) with reference to CLSI, EUCAST or published data. NA, not available

*CCM* curcumin, *CAP* capsaicin, *MET* metronidazole, *VAN* vancomycin, *FDX* fidaxomicin

### Toxin-mediated cytotoxicity of *C. difficile* strains

**Fig. 1:**
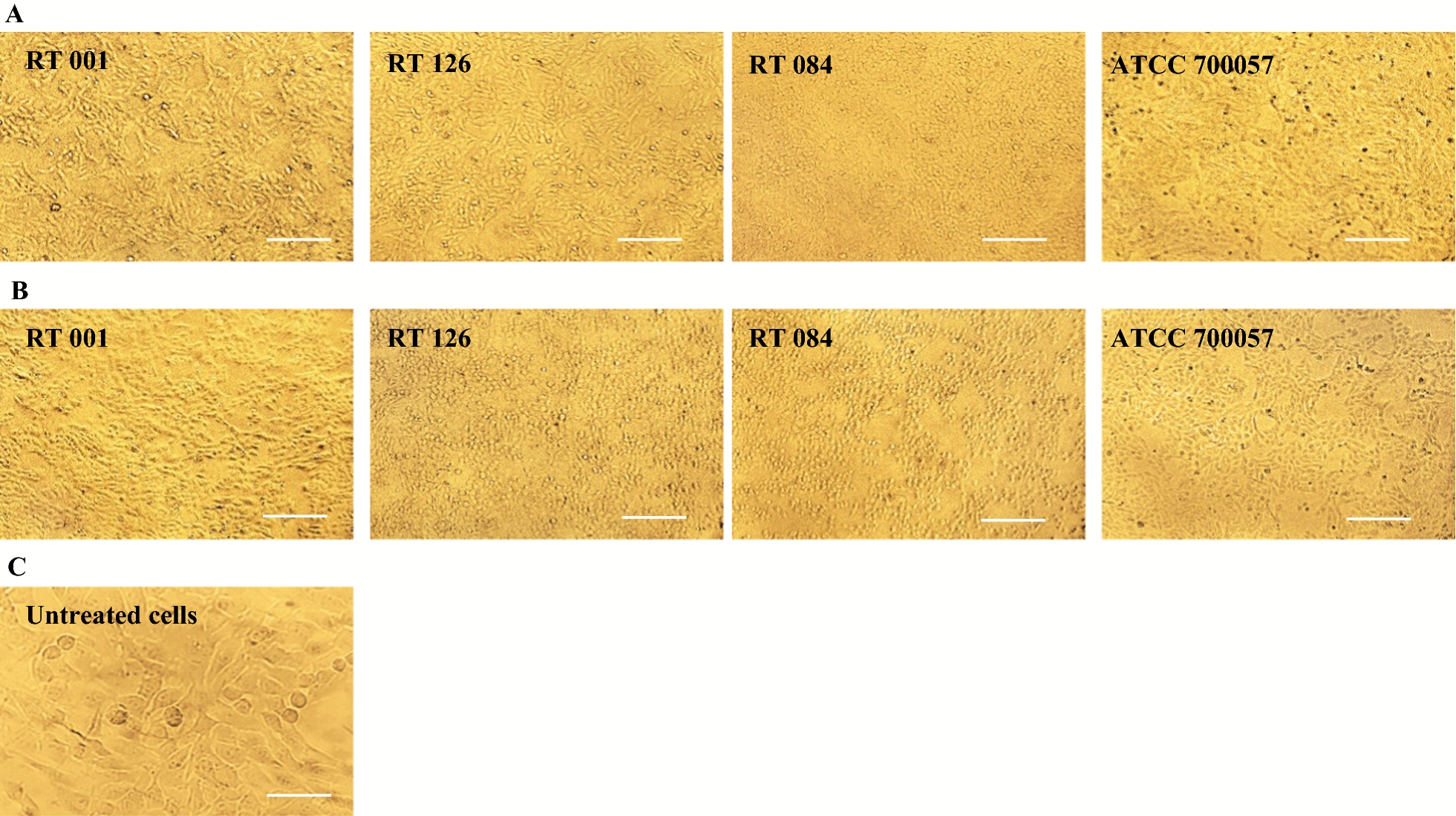
Cytopathic effect (CPE) of two different concentrations (100 and 500 μg/ml) of Tox-S from *C. difficile* (RT 001, RT 126, RT 084) and culture-filtrate of *C. difficile* ATCC 700057 on Vero cells using microscopy. **A** Microscopic cell morphology of Vero cells after treatment with 100 μg/ml Tox-S and culture-filtrate for 4 h at 37 °C; **B** Microscopic cell morphology of Vero cells after treatment with 500 μg/ml Tox-S and culture-filtrate for 4 h at 37 °C; **C** Untreated Vero cell monolayer. **A**, **B** Light microscopy × 20, Scale bar = 50 µm; **C** Light microscopy × 40, Scale bar = 50

To determine the CPE of the four *C. difficile* strains, two different concentrations of Tox-S (100 and 500 μg/ml) and culture-filtrate from non-toxigenic strain were used in cytotoxicity assay using Vero cells. The Tox-S of three toxigenic strains *C. difficile* RT 001, RT 126 and RT 084 caused loss of adhesion, and showed the highest CPE at concentration of 500 µg/ml to achieve 90% cell rounding as compared to culture-filtrate from non-toxigenic strain ATCC 700057 which had no visible effect on Vero cells. The microscopic cell morphology of Vero cells and induced CPE after treatment with Tox-S of the three toxigenic *C. difficile* strains in comparison with culture-filtrate from non-toxigenic strain are shown in Fig. 1.

### Cytotoxicity of CAP and CCM

**Fig. 2:**
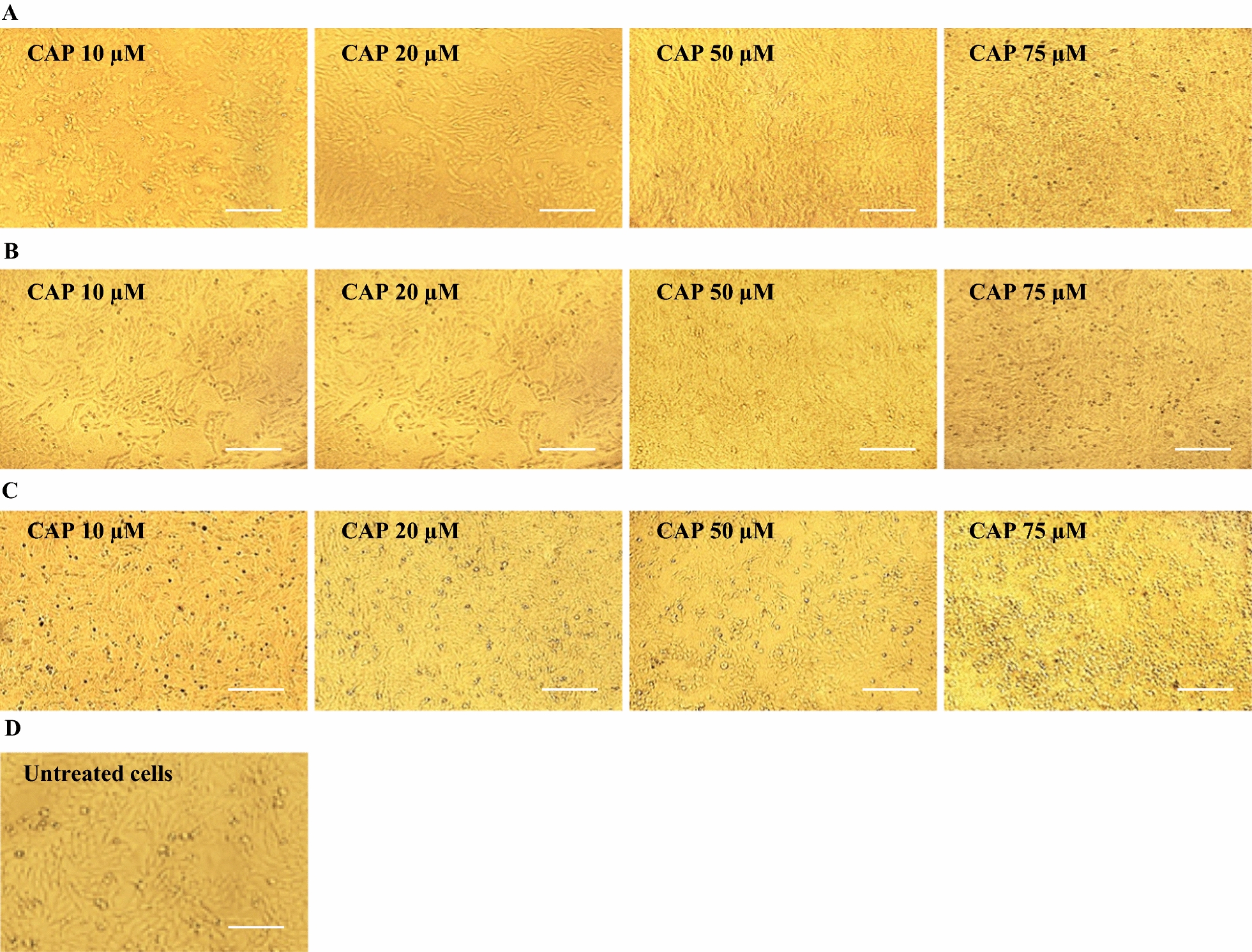
Cytotoxic effect of capsaicin (CAP) on Vero cells. **A** Microscopic cell morphology of Vero cells after treatment with the indicated concentrations of CAP for 1 h at 37 °C; **B** Microscopic cell morphology of Vero cells after treatment with the indicated concentrations of CAP for 4 h at 37 °C; **C** Microscopic cell morphology of Vero cells after treatment with the indicated concentrations of CAP for 24 h at 37 °C; **D** Untreated Vero cell monolayer. Light microscopy × 20, Scale bar = 50 µm

**Fig. 3:**
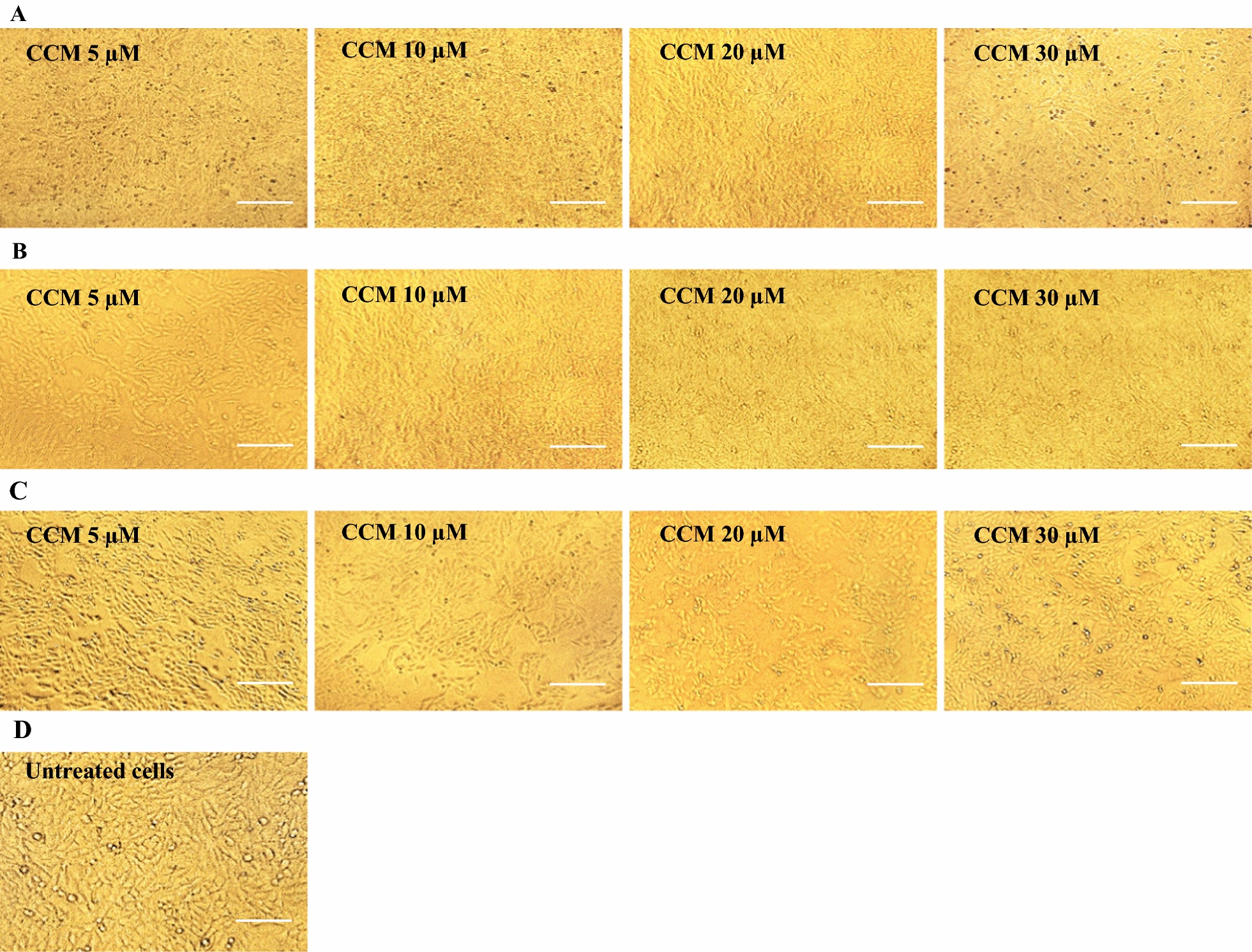
Cytotoxic effect of curcumin (CCM) on Vero cells. **A** Microscopic cell morphology of Vero cells after treatment with the indicated concentrations of CCM for 1 h at 37 °C; **B** Microscopic cell morphology of Vero cells after treatment with the indicated concentrations of CCM for 4 h at 37 °C; **C** Microscopic cell morphology of Vero cells after treatment with the indicated concentrations of CCM for 24 h at 37 °C; **D** Untreated Vero cell monolayer. Light microscopy × 20, Scale bar = 50 µm

To determine the cytotoxic effects of CAP and CCM, Vero cells were incubated with different concentrations of CAP (10, 20, 50 and 75 μM) and CCM (5, 10, 20 and 30 μM) for 1, 4, 24 h. Stimulated Vero cells demonstrated that none of the natural products showed cytotoxic effects after 1 and 4 h. Of the indicated time points, both CAP and CCM showed moderate cytotoxic effects after 24 h of treatment as compared to untreated Vero cell monolayer (Figs. 2 and 3).

### Cell viability of HT-29 cells treated with *C. difficile* strains, CAP and CCM

**Fig. 4:**
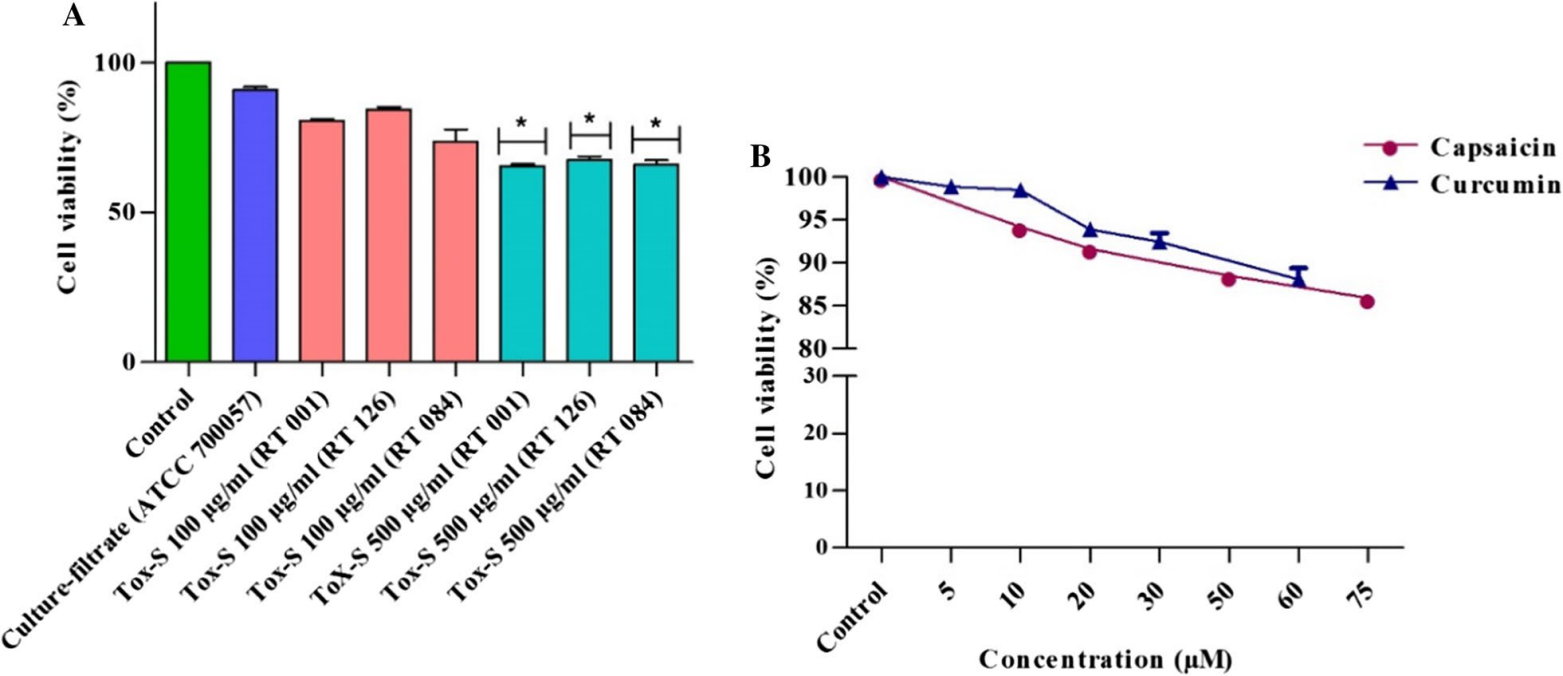
Cell viability determined by MTT assay. **A** Different concentrations (100 and 500 μg/ml) of Tox-S from *C. difficile* (RT 001, RT 126, RT 084) and culture-filtrate of *C. difficile* ATCC 700057 were added to HT-29 cells for 4 h at 37 °C; **B** Different concentrations of capsaicin (10, 20, 50 and 75 µM) and curcumin (5, 10, 20, 30 and 60 µM) were added to HT-29 cells for 24 h at 37 °C. Data were presented as mean ± SD from three independent experiments. A *P* value of < 0.05 was considered as significant (*P < 0.05) by unpaired student’s t test statistical analysis

An MTT assay was performed to determine cell viability of HT-29 cells after exposure to different concentrations of Tox-S of the three toxigenic *C. difficile* strains and culture-filtrate from non-toxigenic strain compared to untreated control cells. As shown in Fig. 4A, only Tox-S of the three toxigenic strains at concentration of 500 µg/ml significantly (*P* < 0.05) reduced the cell viability of HT-29 cells in comparison with culture-filtrate from non-toxigenic strain and untreated control cells. In more details, the Tox-S of toxigenic *C. difficile* strains (RT 001, RT 126 and RT 084) at concentration of 100 µg/ml (80.7%, 84.4% and 73.8%, respectively) and 500 µg/ml (65.5%, 67.5% and 65.9%, respectively) decreased the cell viability of HT-29 cells after 4 h of treatment. The viability of cells after exposure to culture-filtrate from non-toxigenic strain was 91%. The effects of different concentrations of natural products on cell viability of HT-29 cells were also assessed by an MTT assay. The viability of cells after incubation with different concentrations of CAP (10, 20, 50 and 75 μM) and CCM (5, 10, 20 and 30 μM) was 94.1, 92, 88.5, 85.5 and 98.8, 98.5, 93.8, 92.4, respectively. HT-29 cells showed nearly 90% viability after the cells were treated with concentrations of 10, 20, 50 µM and 5, 10, 20, 30 µM for CAP and CCM, respectively (Fig. 4B). Totally, we did not observe any significant reduction in the viability of treated cells with different concentrations of CAP and CCM in comparison with untreated cells (*P* > 0.05).

### *C. difficile* downregulates the gene expression level of BCL-2 and upregulates the expression of SMAD3 and inflammation-related genes in treated HT-29 cells

**Fig. 5:**
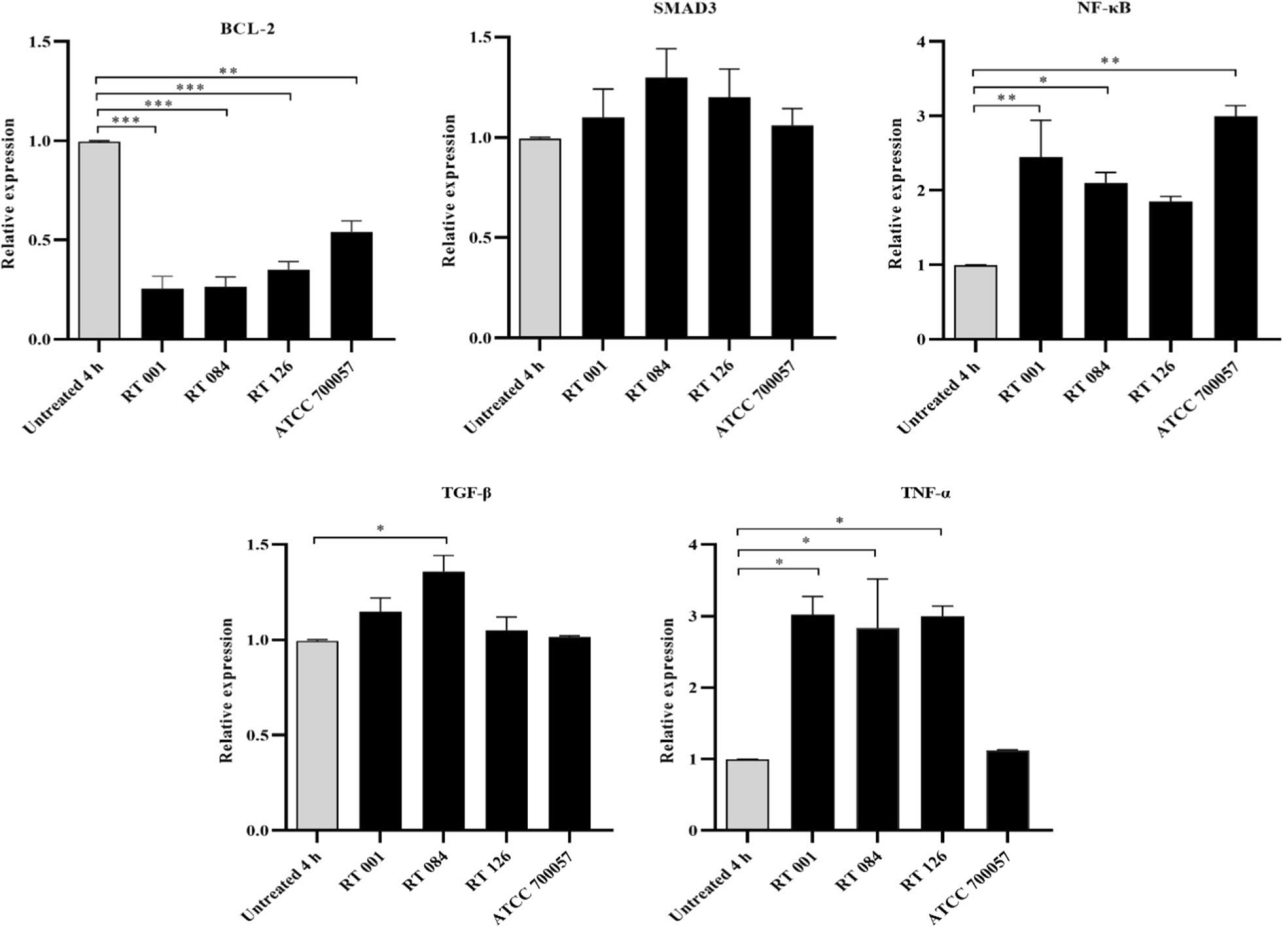
Relative expression of BCL-2, SMAD3, NF-κB, TGF-β and TNF-α genes in HT-29 cells upon treatment with Tox-S (100 μg/ml) from *C. difficile* (RT 001, RT 126, RT 084) and culture-filtrate (100 μg/ml) of *C. difficile* ATCC 700,057 measured by using quantitative real-time PCR assay. Gene expression data were normalized to β-actin as the reference gene. Data were presented as mean ± SD from three independent experiments. A *P* value of < 0.05 was considered as significant (*P < 0.05; **P < 0.01; ***P < 0.001) by unpaired student’s t test and one-way ANOVA statistical analysis

As shown in Fig. 5, treatment of HT-29 cells with Tox-S (100 μg/ml) of *C. difficile* (RT 001, RT 126, RT 084) and culture-filtrate (100 μg/ml) of *C. difficile* ATCC 700057 significantly (*P* < 0.001; *P* < 0.001; *P* < 0.001, *P* < 0.01) reduced the expression level of BCL-2 after 4 h. Although not statistically significant (*P* > 0.05), Tox-S (100 μg/ml) of *C. difficile* (RT 001, RT 126, RT 084) and culture-filtrate (100 μg/ml) of *C. difficile* ATCC 700,057 upregulated the gene expression level of SMAD3. Tox-S (100 μg/ml) of *C. difficile* (RT 001, RT 084) and culture-filtrate (100 μg/ml) of *C. difficile* ATCC 700057 significantly upregulated the gene expression level of NF-κB (*P* < 0.01; *P* < 0.05; *P* < 0.01). Only Tox-S of *C. difficile* RT 084 significantly upregulated the gene expression level of TGF-β (*P* < 0.05). The gene expression level of TNF-α was significantly increased by Tox-S of *C. difficile* (RT 001, RT 126, RT 084) after 4 h of treatment (*P* < 0.05; *P* < 0.05; *P* < 0.05).

### CAP significantly decreased the gene expression level of BCL-2 and SMAD3 in Tox-S treated HT-29 cells

**Fig. 6:**
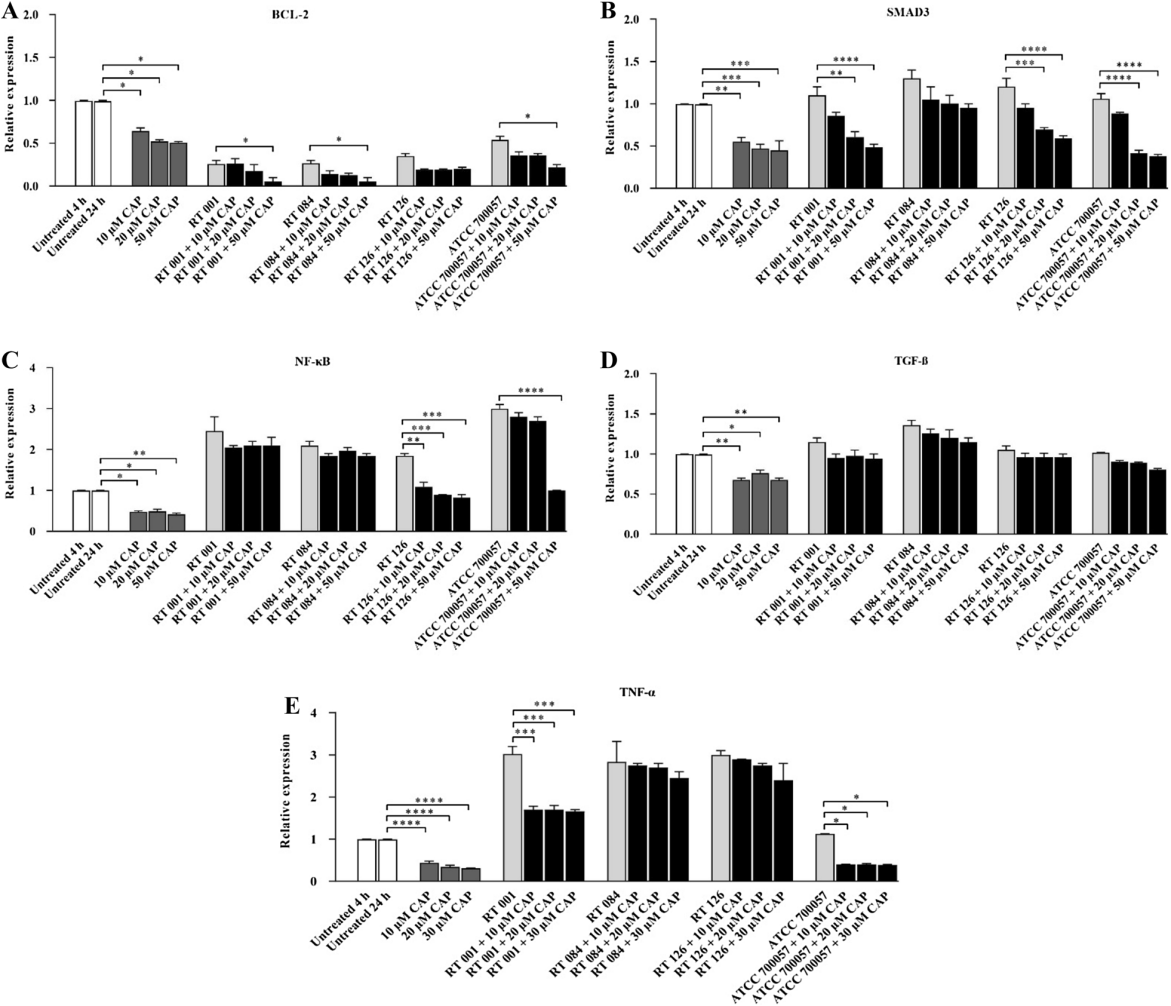
Relative expression of BCL-2 **A**, SMAD3 **B**, NF-κB **C**, TGF-β **D** and TNF-α **E** genes in HT-29 cells upon treatment with Tox-S (100 μg/ml) from *C. difficile* (RT 001, RT 126, RT 084), culture-filtrate (100 μg/ml) of *C. difficile* ATCC 700057, and different concentrations (10, 20, 50 µM) of capsaicin (CAP) measured by using quantitative real-time PCR assay. Gene expression data were normalized to β-actin as the reference gene. Data were presented as mean ± SD from three independent experiments. A *P* value of < 0.05 was considered as significant (*P < 0.05; **P < 0.01) by unpaired student’s t test and one-way ANOVA statistical analysis

The gene expression of BCL-2 and SMAD3 in HT-29 cells were examined upon treatment with Tox-S (100 μg/ml) from *C. difficile* (RT 001, RT 126, RT 084), culture-filtrate (100 μg/ml) of *C. difficile* ATCC 700057, and different concentrations of CAP (10, 20, 50 µM). As shown in Fig. 6A, almost all concentrations of CAP decreased the gene expression of BCL-2 in *C. difficile* stimulated HT-29 cells. In more details, the high concentration of CAP (50 µM) significantly downregulated BCL-2 expression level in HT-29 cells treated with Tox-S of *C. difficile* RT 001 (*P* < 0.05), RT 084 (*P* < 0.05) and culture-filtrate of strain ATCC 700054 (*P* < 0.05). Also, the high concentrations of CAP (20 and 50 µM) significantly reduced the gene expression of SMAD3 in HT-29 cells treated with Tox-S of *C. difficile* RT 001 (*P* < 0.01; *P* < 0.0001), RT 126 (*P* < 0.001; *P* < 0.0001) and culture-filtrate of strain ATCC 700054 (*P* < 0.0001; *P* < 0.0001) as shown in Fig. 6B.

### Effects of CAP on gene expression level of inflammation-related genes in Tox-S stimulated HT-29 cells

To evaluate the effects of CAP on gene expression level of NF-κB, TGF-β and TNF-α, different concentrations of CAP (10, 20, 50 µM) were added to Tox-S stimulated HT-29 cells. As shown in Fig. 6C, CAP used in all different concentrations (10, 20, 50 µM) significantly (*P* < 0.01; *P* < 0.001; *P* < 0.001) reduced the NF-κB gene expression in stimulated HT-29 cells with Tox-S of *C. difficile* RT 126. Also, the highest concentration of CAP (50 µM) was able to significantly (*P* < 0.0001) downregulate the NF-κB gene expression only in stimulated HT-29 cells with culture-filtrate of strain ATCC 700054. As shown in Fig. 6D, almost all different concentrations of CAP (10, 20, 50 µM) slightly decreased the TGF-β gene expression in Tox-S stimulated HT-29 cells, although the differences did not reach statistical significance (*P* > 0.05). Also, all different concentrations of CAP (10, 20, 50 µM) caused statistically significant reductions in TNF-α gene expression of stimulated HT-29 cells with Tox-S of *C. difficile* RT 001 (*P* < 0.001; *P* < 0.001; *P* < 0.001) and culture-filtrate of strain ATCC 700057 (*P* < 0.05; *P* < 0.05; *P* < 0.05) compared with HT-29 cells treated with Tox-S of *C. difficile* RT 084 and RT 126 (Fig. 6E). Moreover, it could be suggested that reduction in TNF-α gene expression is independent of the concentrations of CAP used in this work.

### CCM decreased the gene expression level of BCL-2 and SMAD3 in Tox-S treated HT-29 cells

**Fig. 7:**
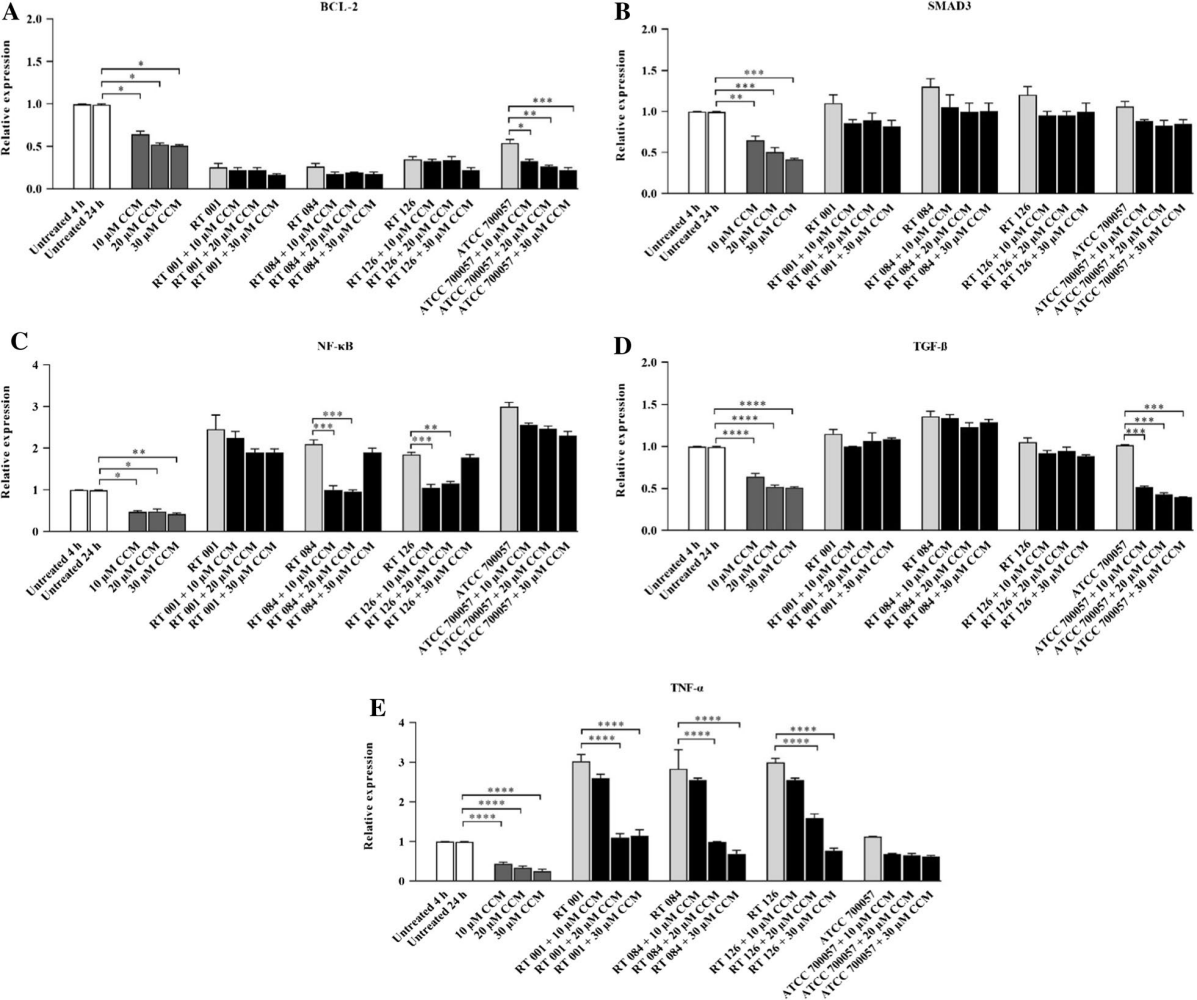
Relative expression of BCL-2 **A**, SMAD3 **B**, NF-κB **C**, TGF-β **D** and TNF-α **E** genes in HT-29 cells upon treatment with Tox-S (100 μg/ml) from *C. difficile* (RT 001, RT 126, RT 084), culture-filtrate (100 μg/ml) of *C. difficile* ATCC 700057, and different concentrations (10, 20, 30 µM) of curcumin (CCM) measured by using quantitative real-time PCR assay. Gene expression data were normalized to β-actin as the reference gene. Data were presented as mean ± SD from three independent experiments. A *P* value of < 0.05 was considered as significant (*P < 0.05; **P < 0.01) by unpaired student’s t test and one-way ANOVA statistical analysis

To examine the effects of CCM on gene expression level of BCL-2 and SMAD3, different concentrations of CCM (10, 20, 30 µM) were added to Tox-S stimulated HT-29 cells. As shown in Fig. 7A, different concentrations of CCM decreased the gene expression of BCL-2 in Tox-S stimulated HT-29 cells. In more details, all concentrations of CCM significantly downregulated the gene expression of BCL-2 in stimulated HT-29 cells with culture-filtrate of strain ATCC 700054 (*P* 0.05; *P* < 0.01; *P* < 0.001). Also, all concentrations of CCM reduced the gene expression of SMAD3 in HT-29 cells treated with Tox-S of *C. difficile* (RT 001, RT 126, RT 084), and culture-filtrate of strain ATCC 700054 as shown in Fig. 7B. However, these reductions were not statistically significant (*P* > 0.05).

### Effects of CCM on expression level of inflammation-related genes in Tox-S treated HT-29 cells

The effects of different concentrations (10, 20, 30 µM) of CCM on gene expression of TNF-α, NF-κB, and TGF-β were also investigated in HT-29 cells treated with Tox-S of toxigenic strains and culture-filtrate of the non-toxigenic strain. As shown in Fig. 7C, almost all different concentrations of CCM decreased the NF-κB gene expression in Tox-S stimulated HT-29 cells. Of note, lower concentrations of CCM (10 and 20 µM) significantly downregulated the gene expression NF-κB in HT-29 cells stimulated with Tox-S of *C. difficile* RT 084 (*P* < 0.001; *P* < 001) and RT 126 (*P* < 0.001; *P* < 0.01). Similarly, CCM decreased the gene expression of TGF-β in Tox-S stimulated HT-29 cells (Fig. 7D). However, these reductions were not significantly different (*P* > 0.05), except for HT-29 cells treated with culture-filtrate of strain ATCC 700054 (*P* < 0.001; *P* < 0.001; *P* < 0.001). Also, all different concentrations of CCM decreased the TNF-α gene expression in Tox-S and culture-filtrate of strain ATCC 700054 stimulated HT-29 cells. As shown in Fig. 7E, the higher concentrations of CCM (20 and 30 µM) significantly decreased the TNF-α gene expression in HT-29 cells stimulated with Tox-S of *C. difficile* RT 001 (*P* < 0.0001; *P* < 0.0001), RT 084 (*P* < 0.0001; *P* < 0.0001) and RT 126 (*P* < 0.0001; *P* < 0.0001).

## Discussion

**Fig. 8:**
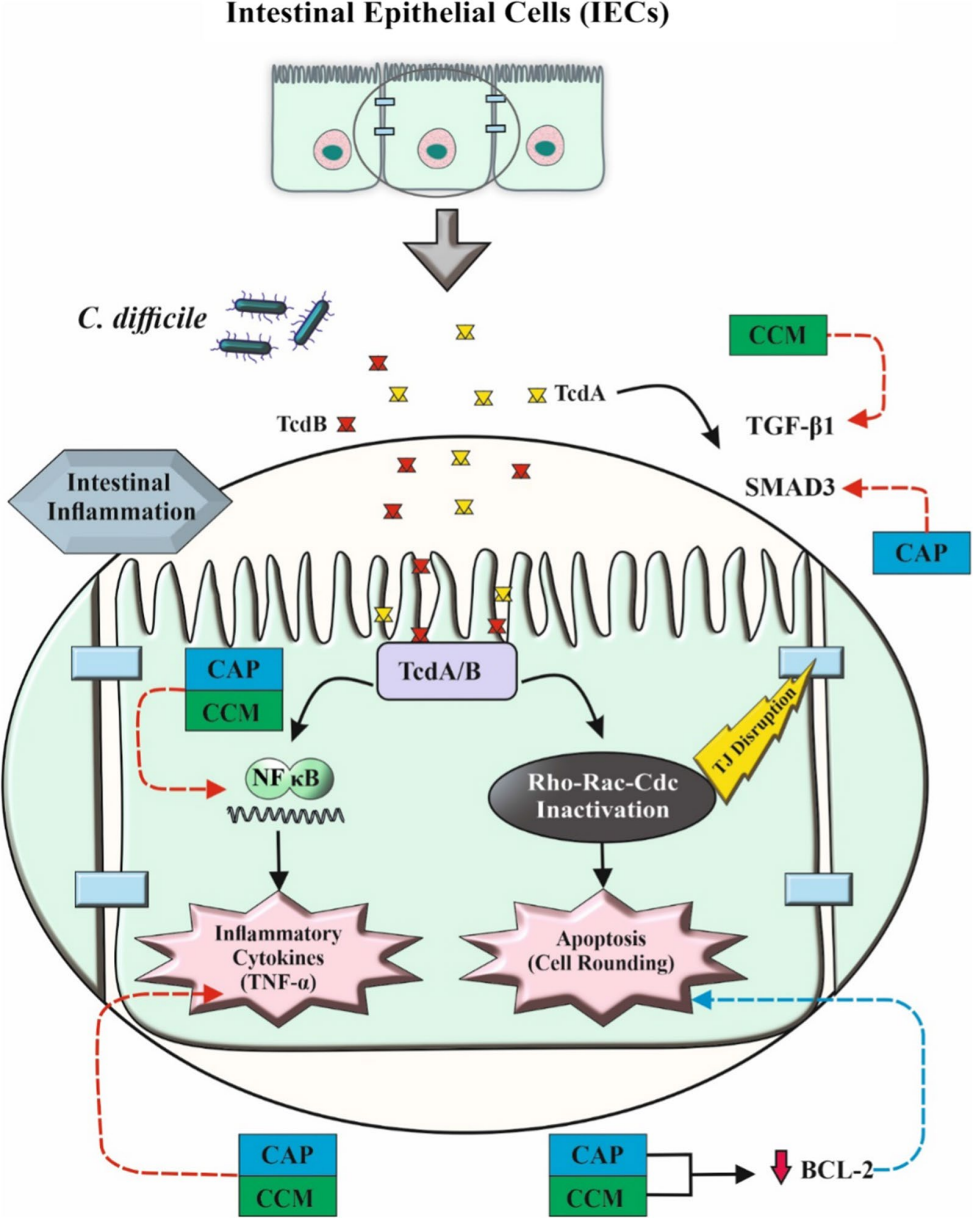
A schematic diagram which demonstrates the potential biological impact of capsaicin (CAP) and curcumin (CCM) on *C. difficile* toxin-mediated inflammation. Exposure of intestinal epithelial cells (IECs) to CCM and CAP can lead to inhibition of TGF-β1 and Smad3, respectively, which may result in maintaining the integrity of the intestinal epithelial tight junctions (TJs) barrier. In addition, CAP and CCM can alleviate host inflammatory responses induced by TcdA and TcdB toxins probably through downregulation of NF-κB and TNF-α. On the other hand, they may induce apoptosis via downregulation of BCL-2 as an anti-apoptotic factor. Notes: red arrows indicate inhibitory actions, whereas blue arrows indicate enhancing actions

The two major toxins, TcdA and TcdB, have long been identified as the primary virulence factors of *C*. *difficile*, which are also associated with the inflammatory response induced during CDI [7]. These exotoxins disrupt host cell function and induce mucosal inflammation through inactivation of small GTP-binding proteins including Rho, Rac, and Cdc42 [54] (Fig. 8). Because of the scarcity of anti-clostridial options and rapid rise in emerging hypervirulent *C. difficile* isolates, the management of CDI has become a global healthcare challenge. Accordingly, exploring novel complementary and alternative therapeutic agents is of great interest in either treating or preventing of CDI [54–56]. So far, a few studies have been carried out to examine the effects of inhibitory properties of CCM and CAP on *C. difficile* toxin activity [41, 42, 57]. Hence, the present study addressed the effect of natural products, CCM and CAP, on toxin-mediated cytotoxicity of *C. difficile* using HT-29 cells treated with Tox-S and culture-filtrate of toxigenic *C. difficile* strains (RT 001, RT 126, RT 084) and a non-toxigenic control strain ATCC 700057. As shown by the agar dilution assay in this study, both CAP and CCM showed an MIC of 256 μM against *C. difficile* RT 001 and RT 126, while MIC 128 μM of CAP and CCM inhibited the growth of *C. difficile* RT 084 and *C. difficile* strain ATCC 700057. These results are relatively in agreement with the study performed by Roshan et al., where they showed that both powder and tablet forms of turmeric exerted a minimal anti-clostridial activity of ≥ 150 µg/ml against five *C. difficile* strains including ATCC 700057, ATCC 43598, RT 027, RT 014 and RT 017 [41]. On the contrary, in a study by Mody et al. different bioactive components of CCM were more effective than FDX in growth inhibition of *C. difficile* strains at concentrations ranging from 4 to 32 µg/ml [57]. These controversial results can be due to different strains of *C. difficile* examined with varied resistance potency in each study. Moreover, this inconsistency may arise from the difference in the nature of phytoconstituents tested, as the activity of each component was varied in the later study. In addition, and to the best of our knowledge, no published studies have previously shown the inhibitory growth effect of CAP on *C. difficile*. Based on our results, CAP can inhibit the growth of different *C. difficile* clinical strains at concentrations ranging from 128 to 256 μM. However, further research using a larger cohort of clinical strains is required to decipher the growth inhibitory action of CAP on *C. difficile* bacteria.

Apoptosis, also known as programmed cell death, is an essential cell process which controls various cellular activities such as the cell turnover and immune response function [58]. A number of proteins, in particular anti-apoptotic B-cell lymphoma 2 (BCL-2) family members, have long been known as an apoptosis suppressor [59]. It has been well established that both TcdA and TcdB from *C. difficile* strains are able to induce cell apoptosis in vitro and in vivo.[11]. In addition, and in consistent with the current concept that toxin induces apoptosis in epithelial cells [60, 61], our treatment experiments indicated that mRNA expression level of BCL-2 was notably decreased upon treatment of HT-29 cells with Tox-S of toxigenic strains and culture-filtrate of *C. difficile* ATCC 700057. However, Tox-S from toxigenic strains could more strongly decrease the gene expression level of BCL-2 in HT-29 cells when compared to untreated control. It is now established that both toxins A and B provoke a complex cascade of host cellular responses and are ultimately responsible for clinical symptoms, inflammation and tissue necrosis [62]. However, it is increasingly appreciated that non-toxin virulence factors likely play essential roles in *C. difficile* colonization, maintenance in the intestinal tract and promotion of potent pro-inflammatory activities [63]. This apoptotic effect can be recognized by cell rounding phenotype in cultured cell lines as observed in our study. CCM is a well-known anti-proliferative component [28] that exhibits an apoptotic effect similar to the toxin A and B of *C. difficile* [7, 10]. It has been reported that CCM can promote the apoptosis cascade particularly through the downregulation of BCL-2 in different cancer cell types [64, 65]. CAP, with a structure similar to that of CCM [66], has several physiological and pharmacological effects with different mechanisms of action [30]. Accumulated studies have shown that CAP can trigger apoptosis in a numerous cancer cell type, however its precise molecular mechanism remains to be elucidated [39, 67, 68]. To date, different studies have investigated the role of CCM and CAP in modulating mRNA expression of BCL-2 [65, 69, 70]. Jung et al. showed that CAP reduced the BCL-2 expression and consequently induced apoptosis in SK-Hep-1 cells [23]. Yang et al. also demonstrated that CCM could induce cell apoptosis via decreasing the ratio of anti-apoptotic BCL-2 in varying concentrations of CCM (100 mg/kg, 50 mg/kg, and 25 mg/kg) [65]. However, further in vitro and in vivo investigations are required to precisely understand the relevant mechanisms and signaling pathways by which CCM or CAP promote apoptosis in CDI.

TGF-β is a pleiotropic cytokine that modulates various cellular processes like cell differentiation, apoptosis and immunosuppression [71, 72]. There is clear evidence that *C. difficile* toxin A induces TGF-β1 mRNA expression level in ileal-loop tissues of mice and rat small intestinal epithelial cell lines [71]. Furthermore, it has been reported that treatment of T84 human colon adenocarcinoma cell line with low concentration of TcdA (< 10 ng/ml) enhanced the mRNA expression of TGF-β1. This effect was associated with induction of TGF-β1 and its receptor, TβRII, phosphorylation and nuclear translocation of SMAD2/3 in both in vitro and in vivo experiments [72]. These findings strongly suggest that TcdA induces activation of the TGF-β1 pathway through canonical SMAD signaling [71]. The current study showed that strains RT 084 (TcdA^+^B^−^) induced the highest expression level of TGF-β1 and SMAD3 in comparison with the other strains. The higher expression level of TGF-β1 and SMAD3 by RT 084 could be owing to overproduction of TcdA in this strain or may be due to the existence of other virulence metabolites in its extracted Tox-S. In addition, RT 084 strains were reported to be more prevalent amongst patients with antibiotic-associated diarrhea and CDI in Algeria, Ghana and Iran [73–75]. This finding highlights the significance of other putative virulence factors in the pathogenesis of various *C. difficile* strains that remains to be elucidated. Results from this study as well as previous studies [71] suggested that this signaling pathway might play a protective role against the effect of toxin-mediated cytotoxicity of *C. difficile* (Fig. 8). NF-κB is one of the well-known regulatory factors that controls transcription of various proinflammatory cytokines and chemokines involved in intestinal immune system [76]. Several studies demonstrated that TcdA and TcdB induce remarkable immune response and stimulate the release of inflammatory cytokines in the gut [77, 78]. Li et al. showed that TcdB significantly activates MAPKs, NF-κB and subsequently induces production of IL-1β and TNF-α [79]. Our data showed that all different *C. difficile* RTs induced the gene expression of NF-κB and TNF-α in treated intestinal HT-29 cells as compared to non-infected control cells. Interestingly, culture-filtrate of non-toxigenic *C. difficile* ATCC 700057 significantly induced the expression level of NF-κB similar to toxigenic strains used in this work. According to a previous study, proteomic analysis of *C. difficile* revealed that several other virulence factors such as extracellular proteases, surface layer proteins (SLP) A, cell wall-binding protein (CWP), collagen-binding protein (CbpA), fibronectin-binding protein (FBP) type IV pili and flagella can also contribute to disease severity and host colonization [80–83]. These bacterial factors possess potent pro-inflammatory activities, and are capable of causing intestinal mucosal injury by promoting inflammatory responses [80, 84–86]. For instance, recognition of *C. difficile* flagellin by toll-like receptor 5 (TLR-5) results in activation of NF-κB and MAPKs pathways, which in turn can induce the production of additional growth factors and cytokines [87]. Therefore, this finding once again highlights the clinical significance of non-toxigenic strains and could shed light on the role of non-toxin virulence factors in the pathogenesis of *C. difficile*. According to several previous studies, CCM has anti-inflammatory and also anti-fibrosis effects by suppressing the NF-κB and TGF-β1/SMAD-3 signaling pathways, respectively (Fig. 8) [88–90]. Vasanthkumar et al. demonstrated that CCM and CAP alone or in combination cause considerable reduction in LPS-induced overexpression of COX-2, IL-6 and TGF-β [91]. Consistent with previous studies [92, 93], findings of the present work revealed that CCM could decrease the *C. difficile*-induced gene expression level of NF-κB, TGF-β1/SMAD-3 and TNF-α in HT-29 cells stimulated by *C. difficile* strains. Moreover, Jingshuang et al. demonstrated that capsaicin reduced the secretion of inflammatory cytokines and TNF-α by inhibiting the NF-κB, and thereby reduced LPS-induced inflammatory response in macrophages [94]. Sugiyama et al. also showed that CAP attenuates TGFβ2-induced epithelial-mesenchymal-transition and reduced TGFβ2-induced p-Smad2/3 in lens epithelial cells in vivo and in vitro [95]. In the same manner, CAP was able to reduce the gene expression level of NF-κB and TGF-β1/SMAD-3 in the present study, however further study is needed to investigate its potential anti-inflammatory effect.

## Conclusion

In conclusion, the results of the present study indicated that both CCM and CAP can modulate the mRNA expression of BCL-2, TGF-β, SMAD3, NF-κB, and TNF-α in HT-29 cells stimulated by different clinical *C. difficile* strains in vitro. Also, we demonstrated the growth inhibitory potential of these two natural products, and which can be utilized as anticlostridial agents to ameliorate *C. difficile* toxin-induced inflammation. The analysis of biological pathways and networks associated to herbal medicines is complicated due to complexity of their derivatives and targets. Further research exploring the inhibitory effect of CCM and CAP and their related components on other key virulence attributes expressed by various *C. difficile* isolates, on *C. difficile* endospore production, germination and outgrowth, and their activity in vivo are worth to be performed. Taken together, ease of availability and affordability of CCM and CAP can introduce them as potential supplements to current treatments used for refractory *C. difficile* infections. However, further studies are needed to accurately explore the mechanistic basis for the anti-toxin activity of such natural components, their beneficial effects, safety and also possible adverse risks in clinical practice through pre-clinical and/or clinical studies.

## Data Availability

No applicable.

## Abbreviations

ANOVA: One-way analysis of variance
BCL-2: B-cell lymphoma 2 family members
BHI: Brain heart infusion broth
CAP: Capsaicin; CCM: curcumin
CCFA: Cycloserine-cefoxitin-fructose agar
CDI: Clostridioides difficile infection
CPE: Cytopathic effect
DMSO: Dimethyl sulfoxide
ELISA: Enzyme-linked immunosorbent assay
FDX: Fidaxomicin
IBRC: Iranian Biological Resource Center
IECs: Intestinal epithelial cells
LCGT: Large clostridial glycosylating toxin
MDR: Multidrug resistant
MET: Metronidazole
MIC: Minimum inhibitory concentration
MTT: 3-(4,5 Dimethylthiazol-2-yl)-2,5-diphenyl tetrazolium bromide
OD: Optical density
RT-qPCR: Quantitative real-time PCR
rCDI: Recurrence rate; rpm: revolutions per minute
SD: Standard deviations
SDW: Sterile distilled water
TcdA: Toxin A
TcdB: Toxin B
Tox-S: Toxigenic cell-free supernatants
TRPV1: Transient receptor potential vanilloid subfamily member 1
VAN: Vancomycin
